# Modeling spatial accessibility to parks: a national study

**DOI:** 10.1186/1476-072X-10-31

**Published:** 2011-05-09

**Authors:** Xingyou Zhang, Hua Lu, James B Holt

**Affiliations:** 1 Centers for Disease Control and Prevention (CDC), Atlanta, GA, USA

## Abstract

**Background:**

Parks provide ideal open spaces for leisure-time physical activity and important venues to promote physical activity. The spatial configuration of parks, the number of parks and their spatial distribution across neighborhood areas or local regions, represents the basic park access potential for their residential populations. A new measure of spatial access to parks, population-weighted distance (PWD) to parks, combines the advantages of current park access approaches and incorporates the information processing theory and probability access surface model to more accurately quantify residential population's potential spatial access to parks.

**Results:**

The PWD was constructed at the basic level of US census geography - blocks - using US park and population data. This new measure of population park accessibility was aggregated to census tract, county, state and national levels. On average, US residential populations are expected to travel 6.7 miles to access their local neighborhood parks. There are significant differences in the PWD to local parks among states. The District of Columbia and Connecticut have the best access to local neighborhood parks with PWD of 0.6 miles and 1.8 miles, respectively. Alaska, Montana, and Wyoming have the largest PWDs of 62.0, 37.4, and 32.8 miles, respectively. Rural states in the western and Midwestern US have lower neighborhood park access, while urban states have relatively higher park access.

**Conclusions:**

The PWD to parks provides a consistent platform for evaluating spatial equity of park access and linking with population health outcomes. It could be an informative evaluation tool for health professionals and policy makers. This new method could be applied to quantify geographic accessibility of other types of services or destinations, such as food, alcohol, and tobacco outlets.

## Background

The rapidly increasing prevalence of obesity and overweight in last two decades has become a dominant public health problem in the United States (US). According to the National Health and Nutrition Examination Survey (NHANES), the estimated age-adjusted obesity prevalence has increased from 14.5% in 1976-1980 to 33.8% in 2007-2008 among adults age 20 years and older in the United States[1-3]. The current obesity epidemic has become a significant contributing factor of several leading causes of mortality and morbidity, including heart disease, stroke, diabetes and some cancers. The estimated healthcare costs of obesity in the US are as high as $147 billion[4]. If the prevalence of obesity remains unchanged, per capita spending on health care for adults would rise by 65 percent by 2020[5]. Both population disease burden and healthcare costs highlight the urgent need to increase population physical activity and healthy eating. An emerging field of study, the impact of the built environment on high energy intake and sedentary behaviors, has gained increased attention[6]. Promoting physical activity is a critical public health strategy to contain the current obesity epidemic and to prevent the occurrence of major chronic diseases. Shaping or improving the local built environment to better support healthy behaviors, such as physical activity, has a potential long-term effect on population health and could be a key avenue for successful obesity prevention[7].

Neighborhood parks are critical components of the neighborhood built environment context, especially in urbanized areas. Neighborhood parks provide ideal open spaces for leisure-time physical activity and important venues to promote population-level physical activity[8]. Lack of, or limited, park access can constrain the physical activity opportunities of local residents, which may increase the prevalence of obesity. Having good access to green space, especially parks, in urban areas is associated with increased physical activity [9,10]. The Task Force on Community Preventive Services recommended creating or enhancing access to places for physical activity as one of eight identified strategies to increase physical activity [11]; and parks are the most common places for local populations for outdoor physical activities[12]. Parks are one of eight societal sectors to promote physical activity recommended by the U.S. National Physical Activity Plan http://www.physicalactivityplan.org/theplan.htm.

However, a review of the research of the relationship between neighborhood park access and residents' physical activity outcomes showed mixed results[13]. The reasons for these inconsistencies could be complex and various across studies. Park access and physical activity could be very different by gender and age groups[14] as well as socioeconomic status[15]. But different methods that quantify/measure the neighborhood park access in previous studies at least contribute to these inconsistencies, since measuring park access is critical to evaluating the effects of park use on physical activity. Park access, by nature, is a multiple dimension concept, at least including: park proximity to neighborhoods (location), park size, park safety, and park attractiveness (amenities, and facility types, quality and quantity). This complicates the methods and the contents to measure park access.

From a geographical perspective, the spatial configuration of neighborhood parks, the number of parks and their spatial distribution across neighborhood areas or local regions, represents the basic park access potential for local residential populations. Thus, it is not surprising that the spatial accessibility of neighborhood parks, mainly based on park proximity and location and size, is commonly used to evaluate the contribution of parks to physical activity in most studies. From a public health intervention perspective, spatial accessibility of neighborhood parks lays the foundation to inform and evaluate further needs for park environment improvement in terms of safety, amenities and/or facilities, and even the need for new parks in some neighborhood areas.

There are an increasing number of studies that examine the linkage between park access and physical activity behaviors. A recent multiple-site study of public park and objectively-measured physical activity for adolescent girls in the US shows that park access (the presence and density of parks in local neighborhoods and proximity to neighborhood parks) was associated with higher levels of nonschool moderate-to-vigorous physical activity[16]. Thus, the spatial accessibility of parks is relevant to physical activity. Developing park access measures is important because they are critical to establishing key correlates and determinants that drive physical activity and inform intervention strategies. However, there is no national existing study that evaluates the potential spatial accessibility of parks in the US. Thus, we aim to create a national database for a neighborhood park spatial accessibility index that could objectively quantify the spatial distribution of neighborhood parks based on residential proximity to parks as well as park sizes; could provide a basic platform for further park access equity inquiry as well as studies of health and health behavior outcomes; and could be used for specific local as well as nationwide studies.

### Methods for spatial accessibility measurement

Current methods for measuring spatial accessibility of neighborhood parks in the literature can be categorized into three general approaches: 1) spatial proximity to parks, which measures travel costs in overcoming spatial separation between the locations of population and parks; 2) the container approach, which measures the existence or density of parks in a defined geographic area; and 3) the spatial interaction modeling approach, commonly known as gravity model-based approach, which measures the potential spatial accessibility of parks. In this paper, we proposed a new approach to measure potential spatial access to parks: population weighted distance. In this paper, we did not consider park accessibility incorporating both space and time dimensions as those space-time accessibility measures in some planning and transportation studies [17,18].

### The travel cost approach

The travel cost approach includes some simple intuitive measures, such as the distance from residential neighborhood to the nearest park or the minimum travel time to the nearest park. These direct (Euclidean or network) distance measures of park accessibility are intuitive and also convenient to generate in a geographic information systems (GIS) environment. The major problem of this approach is that it assumes residents would always use the nearest park with the least travel cost as a space for physical activity. The exclusive use of one nearest park by local neighborhood residents is not realistic. A modified distance measure goes to another unrealistic extreme, which takes the average distance from an origin (home or residential neighborhood) to all its potential park destinations to measure spatial proximity to parks[19,20].

### The container approach

A more common approach for measuring park accessibility used in previous studies is the container-based method. In this method one summarizes the number of parks, or the total area of parks within a geographic unit. This geographic unit is often defined by the basic neighborhood unit under study, such as census tract, ZIP code, or local neighborhood unit; this geographic unit could also be defined by the area within the specified walking distances from residential locations. Specific measures of spatial accessibility of parks include a simple indicator of the existence of parks within the defined geographic unit, the number of parks, the total park area size within a neighborhood or within walking distance buffers. The percentage of land area used for parks per neighborhood, as well as the total area of park space averaged by population size are commonly used measures in park access equity analysis.

However, there are several problems associated with these container-based accessibility measures. First, the container-based measures are subject to a well known spatial analysis problem: the Modifiable Areal Unit Problem (MAUP). In geographic studies, MAUP means geographic measures or relationships of interest could change because of the definition of spatial scales of the geographic unit of analysis. The spatial accessibility of parks may change depending on the spatial size (extent) of geographic containers. So a neighborhood or residential place could have very different park accessibility measures, depending on the size of the geographic container. For example, a quarter mile (400 meters), half mile and one mile are often used for walking distance to create a buffer area (geographic container) for highly-urbanized areas to define spatial accessibility of parks; and these differences in walking distances to define the geographic container could lead to inconsistencies in park accessibility measures for the same neighborhood or residential places. For most suburban and rural populations, cars may be used to access local parks. The concept of neighborhood parks thus could be very different from those inside the central cities. Neighborhood parks were defined as outdoor public areas within 10 miles or a 20-minute drive from residents' locations [21]. This will make the definition of geographic container very difficult. Also, larger sizes of geographic containers could result in a serious ecological fallacy problem that population within a neighborhood share the same spatial accessibility of parks. Another obvious problem with a container approach is edge effects. A defined neighborhood or a neighborhood with buffer areas may have no parks inside but may have some or more outside ones near its boundary, but this neighborhood assumes no access to these parks. Thus, the traditional container-based measures could be very biased indicators and could create some unrealistic areas that have no access to parks at all[22].

Kernel density estimation, an improved container method, was recently applied to better characterize the spatial accessibility to neighborhood parks [23]. The kernel density estimation method assumes that the park accessibility will decrease from the park location to its neighborhood areas within a threshold distance (called kernel bandwidth). The kernel density function determines how the value of park accessibility decreases from the peak value at park location to zero at the threshold distance. Kernel density estimation has two basic components: kernel functions and kernel bandwidth. The choice of kernel bandwidth usually has much stronger effects on park accessibility density surface than the choice of kernel functions. The larger the distance for kernel bandwidth, the smoother the resulting park accessibility surface. Each park has a spatial accessibility density surface. A neighborhood's park accessibility is the sum of all park accessibility density surfaces that are covering this neighborhood. If a neighborhood is not located within any park kernel zones, its park accessibility would become zero. How to select an appropriate kernel bandwidth is always challenging and adaptive kernel density may be a better choice[24]. Obviously kernel bandwidth strongly depends on the spatial behaviors or processes under study. In park accessibility studies, one-mile or quarter-mile walking distances are often used as the kernel bandwidth; but this will leave many neighborhood areas without any access to parks. So by nature, this is a modified container approach. Compared to traditional container-based measures, the kernel density park accessibility measure overcomes the assumption of equal accessibility within the container and could quantify the heterogeneity of park accessibility within it.

### Spatial interaction modeling approach

Another important method for measuring geographic accessibility is the spatial interaction model, which is an extension of a gravity model (here we assume these two terms are exchangeable). This approach assumes that the spatial interaction declines with a larger spatial separation (travel distance or time) between origins and destinations; spatial interaction increases with a greater demand at origins or with higher supply capacity and/or attractiveness at destinations. In the context of park access, the spatial interaction (*A*_*ij*_) from a residential place (*i*) to a destination park (*j*) is defined as(1)

where *S*_*j *_is the destination park size *j*, *d*_*ij *_is the distance from a residential neighborhood *i *to destination park *j*, *α *and *β *are the parameters associated with park size and distance respectively and measures their effects on park accessibility. The potential spatial accessibility index *A*_*i *_for a residential neighborhood *i*, is defined as the sum of spatial interaction (*A*_*ij*_) with all its destinations parks:(2)

*A*_*i *_is used to measure the potential park accessibility from a residential place to its neighboring parks. Thus, residential neighborhoods with shorter distances to parks will have higher potential park accessibility; similarly, parks with a larger size will attach more residents. Besides the large physical space, larger parks usually have more facilities and public service programs that could attract more local residents[25].

There are two theoretical advantages for the spatial interaction model-based accessibility measures. First, the spatial interaction model approach avoids a significant problem of the container-based approach, which requires that a neighborhood or a local geographic area has to be defined to generate park accessibility measures. The spatial extent of parks for a local neighborhood could vary according to different geographic settings (inner city, suburban and rural areas) and it completely depends on the spatial distribution of parks for a specific neighborhood. Without the concerns of defining neighborhood size, this approach would minimize the influence of MAUP on accessibility measures. Second, this approach could easily generate more-accurate localized population exposures to parks, which will reduce the ecological bias of park accessibility measures and the influence of an ecological fallacy for population park access measurement. When linked to individual health outcomes, park accessibility measures could be based on accurate individual home locations or residential census blocks, the smallest census geographic unit in the US.

Spatial interaction measures have been developed and used for numerous studies on urban facilities or services, however only a few studies [26-29] employed them for measuring park accessibility. Besides park size and distance to parks, these studies also incorporate the park quality or attractiveness in their park accessibility measures in the form , where  is the attractiveness measure of park *j *with an influence parameter *λ*. It highlights another methodological advantage: the spatial interaction modeling framework could flexibly incorporate other park features that may affect park access and use, such as park safety, quality, and facilities, when these information or measures are available.

#### Distance decay in spatial interaction modeling

Any approach to measuring park accessibility will have its own limitations. There are two common methodological drawbacks associated with spatial interaction accessibility measures: distance decay parameter and spatial destination choice set. The first is the choice of the magnitude of parameter associated with distance, often called distance decay or friction parameter (*β*). Theoretically the distance decay effects on spatial interaction processes or behaviors, reflected by this friction parameter, could be very context-specific, such as the influence of geographic settings (urban, suburban and rural areas) and could vary significantly among different human activities, such as shopping *versus *recreational activities. A larger distance decay parameter indicates that human behavior is more sensitive to distance. The distance decay parameter could even be very different among different types of destinations for the same type of activities. For example, in recreational physical activity behaviors, the distance decay parameter is 1.91 for the public's use of public open space and 1.16 for sporting and recreation centers and golf courses(1.06)[27]. Empirical study is the best way to estimate this parameter for a particular type of human behavior. However the information or data needed to calibrate this parameter usually are not available. Many studies just arbitrarily set a value for this distance decay parameter for the spatial interaction accessibility measures according to their experience or by following some common practice[20]. Biles-Corti and Donovan used the data collected from a social ecological project on environmental determinants of physical activity and explore the distance decay parameters for nine different types of facilities for recreational physical activity in metropolitan Perth, Western Australia. Publically accessible facilities or services usually have larger distance decay parameters than membership or fee-based facilities or services. The distance decay parameter for public open space, such as parks, was estimated at 1.91[27], and this is close to the value of 2.0 which is a commonly used distance decay parameter. In the US, there are no empirical studies that evaluate the parameters for distance as well as park sizes in spatial interaction models. So we adopt this 1.91 as the decay parameter in our spatial interactions of park accessibility analysis.

#### Destination set formation in spatial choice modeling

The second problem associated with spatial interaction accessibility measures is the uncertainty associated with the availability and formation of destination choice sets. Most gravity model-based measures of geographic accessibility say little about the availability and formation of destination choice sets[30]. Destination set formation is not a problem for either container-based measures or nearest distance measures. The destination set is defined as all parks within the predefined geographic areas containing the residential neighborhoods or the nearest park to the residential places. As discussed above, for the container-based measures, all the parks within a container are included in the final access measure computation. When a neighborhood does not have any parks in the defined container, its container-based measures will be zero. For the nearest distance measure, it only counts the nearest park to a neighborhood, and populations only access this nearest park. However, the destination set formation problem arises for spatial interaction model-based measures, since we have to find a set of destinations (e.g. parks) that could interact with the population of a neighborhood. Current spatial interaction measures assume a neighborhood could interact with all the parks in the study area and hence would include all the parks to compute accessibility for this neighborhood. Theoretically, this is not a problem. In the context of park access, a person does have access to all destinations (parks), regardless of where he or she resides in the US. Thus, potential park accessibility should always be greater than zero for all US residential populations. But most parks are mainly used by nearby residents. In reality, a resident population actually uses a much smaller subset of all the parks in a region and even local areas. If all the parks in the study area are included to calculate the potential park spatial accessibility measures of a neighborhood, significant bias could be introduced. So the park destination set should be more than one but less than the full set of parks in a region. Especially in our study, we could not assume that a neighborhood population will interact with all US parks. This is a problem not recognized by most studies which have utilized spatial interaction model-based measures, especially in the context of park access. This is also related to a still unsolved destination set formation problem in spatial choice modeling[31].

The formation of a destination set of parks for a neighborhood population is very complex and could be associated with many demographic and geographic factors. From a psychological perspective, one significant factor is our brain's capacity for information processing. Marketing research shows that individuals have a limited capacity for processing large amounts of information when the choice set of destinations is large[32]. This means the information to make a choice among a set of alternative destinations easily could be beyond the individual's capacity for information processing. This reflects an upper limit on our brain's capacity to process information on simultaneously interacting elements with reliable accuracy and validity[33]. This upper limit is seven plus or minus two: Saaty and Ozdemir mathematically showed that, in order to make consistent preference judgments on pairs of elements in a group, the number of elements in the group should be no more than seven[34]. Our spatial cognition capacity should share this upper limit of seven plus or minus two, as determined from information processing. Our individual limited knowledge (cognition) of spatial alternatives is related to location proximity[35]. Pellegrini et al. empirically examined parameter sensitivity to choice set specification in the context of shopping destination choice and found that model parameters show encouraging stability with relatively small choice sets of seven to ten stores[36]. A choice set of the seven nearest parks may be the possible set that a resident most likely takes into consideration to make a preference decision for park visits with reliable certainty. The choice set of seven nearest parks may be a sample set that evaluates the potential significant correlations between park access and physical activity behaviors as shown in a theoretical analysis of sampling distributions for detection of correlations[37].

### New park accessibility measure: population-weighted distance (PWD)

The spatial interaction accessibility index ( and its various forms) is directly used to quantify the potential spatial access to parks but lacks the intuitiveness of the direct distance measures. Thus, we further introduced the probability access surface from the market penetration approach that assumes the probability of residential population in a neighborhood to visit a park is proportional to its accessibility to this park[38]. We used the spatial interaction accessibility measure as weights for the sampling probabilities of park choice for residential populations and applied the Huff trade area model to compute the probability (*P*_*ij*_) that a resident at a neighborhood (*i*) will choose to visit a park (*j*), as the following formula[38]:(3)

This formula shows residents are more likely to visit nearby parks and larger parks could attract more distant residents. The expected distance (*D*_*i*_) for a neighborhood population (*Pop*_*i*_) to its nearby parks was calculated as the following:(4)

Where *n *is the number of parks that the population in the neighborhood (*i*) are most likely to visit. So *D*_*i *_is the population-weighted distance (PWD) from the neighborhood (*i*) to its nearby parks. This measure has the intuitiveness of direct distance measures, and more importantly, it incorporates the concept of population probability access to parks based on the spatial interaction accessibility index. It allows us to evaluate the population accessibility to parks as well as the potential park service population burden.

In the remainder of this paper, we develop a method that makes use of the advantages of spatial interaction accessibility measures and the choice set of destination parks and probability access surface in order to calculate the PWDs to parks in the US at different geographic levels.

## Methods

### Data

#### 1) Parks

We obtained data on park spaces from the park GIS layer in ESRI ArcGIS9.3 Data DVD (ArcGIS 9.3, ESRI, Redlands, CA). It was created in 2008 with 35,436 public park or forest units in the 50 states and DC. The park dataset includes national, state, and local parks and forests. Park size and within-park centroids were generated in ArcGIS9.3. Very small parks of less than 4000 square feet (or 0.1 acres) are not available in this dataset and are not included in this study.

#### 2) Geographic unit of analysis and population demographics

We selected the census block as our basic geographic neighborhood unit of analysis for two reasons: 1) census blocks are the smallest geographic unit for the US Census. Geographic units in most studies on neighborhood effects on health (e.g., census tracts and ZIP codes) are built upon census blocks. The spatial accessibility of parks for other larger geographic neighborhood units could be easily generated from census block estimates; this also minimizes the bias introduced by MAUP. 2) The interior geometric centroids for census blocks are a more accurate geographic location measure for local population residence than the centroids for census tracts or other larger geographic units. Geographic accessibility measures computed from census tract centroids could yield important measurement errors for 5% to 10% compared to the population-weighted accessibility measures for census blocks within census tracts[39]. Thus, the use of census block centroids provides a more accurate characterization of the population exposure to park spaces and minimizes ecological bias in population locations.

Census block population is not available annually. Therefore we used US census updated 2008 county populations to update census block. 2008 county population estimates by demographics (age, sex, race and Hispanic origin) were downloaded from the US census http://www.census.gov/popest/datasets.html. The 2008 US county populations were allocated to census blocks according to the census block 2000 population demographic composition within a county. This is a limitation but it is preferable to using outdated 2000 census block population data, because the US population landscape has changed significantly since 2000.

Census block group poverty rates in 2008 were obtained from Geolytics http://www.geolytics.com/. We classified the census block groups into three categories according to their poverty rates: low poverty block groups with poverty rates less than 10%; medium poverty block groups with poverty rates equal to or greater than 10% but less than 20%; high poverty block groups with poverty rates equal to or greater than 20%. We used this block group poverty status to explore whether the populations in the poor neighborhoods have lower spatial accessibility to parks compared to those in the more affluent neighborhoods.

We used National Center for Health Statistics (NCHS) six-level urban-rural classification scheme for the 3,141 U.S. counties and county-equivalents to explore the influence of urbanization level of residence on park spatial accessibility. The county level urban-rural continuum category consists of: 1) large central metro, 2) large fringe metro, 3) medium metro, 4) small metro, 5) micropolitan, and 6) non-core rural counties http://www.cdc.gov/nchs/data_access/urban_rural.htm. The NCHS Urban-Rural Classification scheme was developed with an aim to study the association between urbanization level of residence and health outcomes and to monitor the population health of urban and rural residents. It is also a most updated urban-rural classification system.

#### 3) Distance

Distance is a key metric to construct spatial accessibility. Although it could be measured in various ways, two common distance metrics are network distance and Euclidean distance. Network distance is measured by the length of the shortest street network linking an origin and a destination. Euclidean distance is the length of the straight geometric line linking an origin and a destination. Both can be generated in current GIS software. Theoretically, Euclidean distance is always less than network distance. Network distance is considered to be a more accurate approximation of the actual travel distance from an origin to a destination, and it has been employed in most park access studies. In an urban area with relatively high density street networks, Euclidean distances are strongly correlated with more accurate network distances [39] and even more accurate travel time is not more sensitive to spatial accessibility modeling than Euclidean distances[40]. But the differences will become larger when street network density decreases from urban to suburban and rural areas; and using Euclidean distances for measuring accessibility will have more bias. On the other hand, park access often involves the use of cars in suburban and rural areas and in these areas populations should be less sensitive to distance which could reduce the potential bias for the use of Euclidean distance. In this study, for practical reasons, we use the Euclidean distances between census blocks and parks. Both census block and park locations are proxied by their internal centroids. Current GIS can calculate both network and Euclidean distances between all residential locations and all parks for local studies, but they do not allow us to calculate these distances from all census blocks in US (8,205,582) to all parks (35,436). Therefore we calculated the Euclidean distance between census blocks and parks using SAS (version 9.2, SAS Institute, Cary, NC).

### Calculate census block potential access to parks

Spatial interaction modeling, in general, shows that facility utilization is proportional to the facility size and decreases with the distances between individuals and facilities. Thus, the potential spatial accessibility (*A*_*ij*_) from a block *i *to a park *j *is defined as follow:(5)

Where *S*_*j *_is the size of neighborhood park *j *in square miles; *d*_*ij *_is the Euclidean distance between the centroid of the census block *i *and the centroid of the nearest park *j *in miles; *α *is the parameter that reflects the size effects of the nearest park *j *on its accessibility; β is the parameter that characterizes distance decay effects of access to nearby park *j*. Based on the only available study, which is from Australia, *α *has an empirical value of 0.85 and β has a value of 1.91 for public open space [27]. This potential access model is only based on the distance from block to park and on the park size, and we do not include park attractiveness in the model for two reasons. First, park attractiveness is associated with many factors, such as sport and children facilities, presence of walking paths, landscaping, and physical amenities (nearby ocean, river, or lake) and park safety. These detailed data are not available for the nation-wide park dataset. The other more important reason is that the spatial arrangement of parks (location and size) is usually fixed, while most park attractiveness characteristics can be modified. Thus this potential access model provides a basic metric quantifying the spatial distribution of parks and its relationship with nearby neighborhoods. It could provide an answer for the question of whether we need more or better quality parks in a neighborhood.

The total potential accessibility to nearby parks is the sum of the accessibility from the census block *i *to all its nearest seven parks:(6)

### Calculate the probability from census blocks to parks

Each census block has a potential set of destination parks of seven but the probability that census block populations interact with each nearby park should vary. We adopt the equation 3 with the following form:(7)

All the parameters are the same as defined in equation 5 and 6. With this probability model, we could estimate the potential number of census block residents that will visit each nearby park by multiplying the total census block population by the probability that a resident from that census block utilizes each park. More importantly, it allows us to incorporate population spatial heterogeneity into the larger neighborhood unit (e.g. census tract and ZIP code) park access calculation. The weighted sampling of seven nearest parks from a large full set of park alternatives may give a more accurate estimate of true expected travel distance to local parks for residential populations in a neighborhood as indicated in some empirical spatial choice modeling[41].

### Calculate block level PWD to parks

The above potential accessibility index to parks is commonly used in the literature. Higher accessibility scores indicate better accessibility to parks, but these are not intuitive compared to direct distance measures. So we further use this spatial interaction accessibility measure to generate an intuitive distance measure to evaluate the spatial accessibility to parks. Using the probability model above, the block level PWD (*B*_*i*_) to nearest parks for the population in that census block (*Pop*_*i*_) is defined as:(8)

Similarly, the PWD for the population in larger geographic units than census block (block groups, census tract, ZIP codes, counties, states, and even the entire US) to visit nearest parks is defined as:(9)

where *n*_*k *_is the number of blocks in a more aggregated geographic unit (*k*) than a block, and *Pop*_*k *_is the total population of the aggregated geographic unit (*k*), and *T*_*k *_is the PWD for the aggregated geographic unit (*k*) to visit parks. By nature, it is a PWD to nearest parks and measures the overall potential spatial accessibility to parks for the geographic unit of interest.

This PWD itself is as intuitive as the traditional distance measure; it could be viewed as an adaptive container-based measure with a constant choice set of seven nearest parks; but it is based on a flexible spatial interaction-based accessibility index. This compound measure provides us a useful metric to characterize the spatial structure of parks (location, size) and its relationship to residential neighborhoods. It could also be conveniently visualized at different geographic levels (census block, tract, and county) in a GIS environment.

## Results

**Figure 1:**
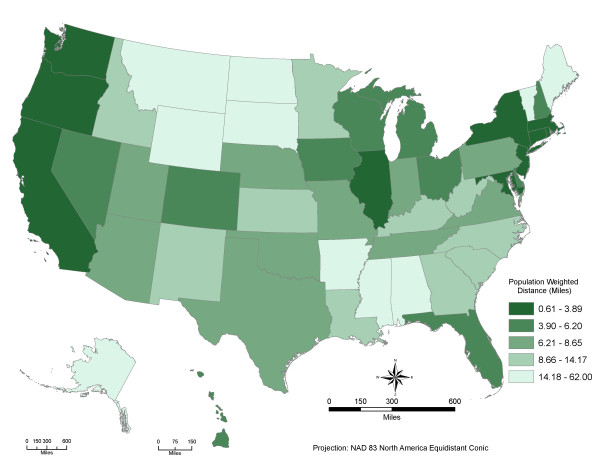
**The population-weighted distances to parks by state in the U.S**. Darker green means better potential spatial access to parks for local residential populations

We calculated the PWDs for the entire US, including 50 states and DC, starting from the census block level and further aggregating to census tract, county, state and nation. Our preliminary results show that people in the US, on average, are expected to travel 6.7 miles to access their local neighborhood parks (table 1). There are significant differences in the PWDs to local parks among states (see Figure 1). The District of Columbia and Connecticut have the best access to local neighborhood parks with the PWDs of 0.6 miles and 1.8 miles, respectively. Alaska, Montana, and Wyoming have the largest PWDs of 62.0, 37.4, and 32.8 miles, respectively. Rural states in the western and Midwestern US have lower neighborhood park access, while urban states have relatively higher park access.

**Table 1:** The population-weighted distances to parks (miles) by urban-rural continuum, race/ethnicity and census block group poverty status in 2008.

Geography	Poverty	ALL	White	Black	Asian	Other	Hispanic
US	ALL	6.7	7.6	5.8	2.5	11.4	4.4
US	LOW	5.9	6.5	5.1	2.4	6.5	4.0
US	MEDIUM	8.9	11.0	7.2	2.8	12.2	4.9
US	HIGH	6.9	9.3	5.5	2.7	22.0	4.6
Large central metro	ALL	1.2	1.3	1.0	1.0	1.2	1.1
large fringe metro	ALL	3.0	3.2	2.4	1.6	2.8	2.3
Medium metro	ALL	6.8	6.9	7.8	3.6	6.5	6.1
Small metro	ALL	14.5	14.3	16.0	13.0	19.3	13.5
Micropolitan	ALL	15.0	14.2	18.3	15.4	19.7	18.4
Noncore	ALL	22.2	20.7	23.5	37.2	46.4	24.2
Large central metro	LOW	1.3	1.4	1.3	1.0	1.3	1.3
Large central metro	MEDIUM	1.1	1.2	1.0	1.0	1.1	1.1
Large central metro	HIGH	0.9	1.0	0.8	0.8	1.0	0.9
large fringe metro	LOW	2.9	3.1	2.4	1.7	2.7	2.4
large fringe metro	MEDIUM	3.7	4.5	2.7	1.6	3.2	2.6
large fringe metro	HIGH	2.4	3.2	2.2	1.2	3.0	1.9
Medium metro	LOW	6.5	6.7	7.7	3.7	5.9	5.2
Medium metro	MEDIUM	7.9	8.4	9.1	3.9	7.5	6.0
Medium metro	HIGH	6.6	6.2	6.9	3.1	7.0	7.1
Small metro	LOW	14.0	13.9	15.7	12.8	16.0	14.0
Small metro	MEDIUM	15.4	15.3	17.0	13.0	19.0	14.4
Small metro	HIGH	14.6	14.5	15.4	13.4	25.8	12.1
Micropolitan	LOW	14.1	13.8	16.6	15.3	16.1	16.0
Micropolitan	MEDIUM	15.8	14.9	19.4	15.2	18.8	18.3
Micropolitan	HIGH	17.1	14.3	18.7	15.8	26.8	21.5
Noncore	LOW	19.7	19.2	21.6	48.0	26.9	22.4
Noncore	MEDIUM	22.2	21.6	23.6	28.7	34.0	22.9
Noncore	HIGH	28.2	23.0	24.4	23.9	65.3	28.8

Table 1 shows that the PWD to local parks varies by neighborhood poverty (low(<10%), medium (> = 10% and <20%) and high(> = 20%) defined by census block group level poverty rates), urbanization levels (large central metro, large fringe metro, medium metro, and small metropolitan areas, micropolitan areas and noncore rural areas) for different population groups (non-Hispanic white, non-Hispanic black, non-Hispanic Asian, Hispanic and other minorities). The PWD is 8.9 miles (highest) for the neighborhoods with medium poverty rates, 5.9 and 6.9 miles for the neighborhoods with high and low poverty rates, respectively. Similar patterns are shared for subpopulations: more affluent neighborhoods enjoy the best park access, and poorer neighborhoods have better park access than neighborhoods with medium poverty rates.

**Figure 2:**
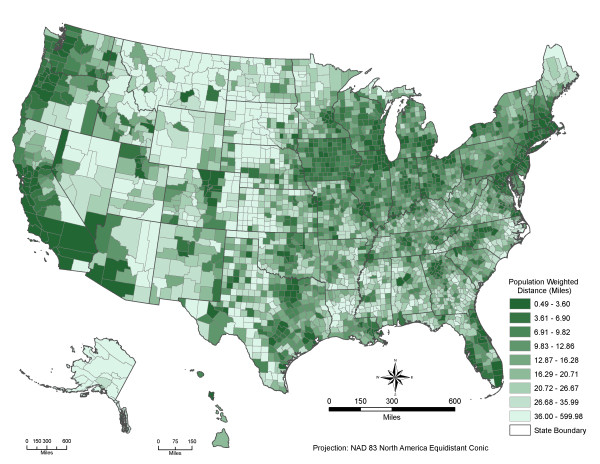
**The population-weighted distances to parks by county in the U.S**. Darker green means better potential spatial access to parks for local residential populations

Among the urban-rural geographic continuum in the US (table 1 and Figure 2), the PWD to local parks is 1.2 miles for large central metropolitan counties, increases to 3.0 miles for large fringe metropolitan counties, 6.8 miles for medium metropolitan counties, 14.5 miles for small metropolitan counties, 15.0 miles for micropolitan counties, and 22.2 for noncore rural counties. Within the spectrum of metropolitan areas, the PWD increases more than two times from a more-urbanized county to a less-urbanized county. Different subpopulation groups follow similar patterns of local park access.

For the racial/ethnic population groups in the US, Asian and Hispanic populations have the smallest PWDs to local parks, 2.5 and 4.4 miles respectively; the PWD is 5.8 miles for black population, 7.6 for non-Hispanic white population, and 11.4 miles (the largest) for other minority population groups.

**Figure 3:**
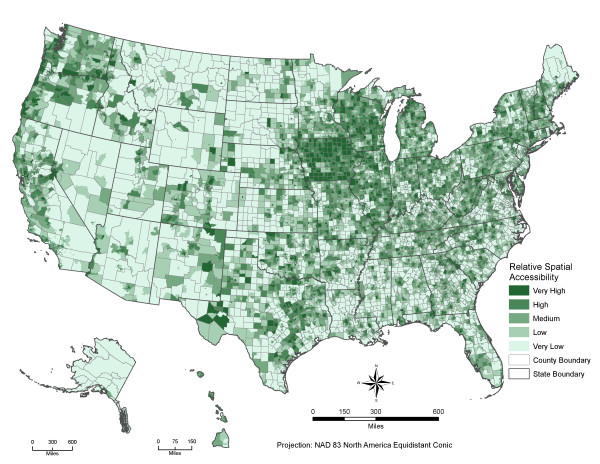
**The PWD-based relative park spatial accessibility to parks by census tract in the U.S**. Darker green means better potential spatial access to parks for local residential populations Note: census tract level PWD was classified into five groups according to their quintiles by NCHS county level urban-rural classification scheme: 1) large central metro, 2) large fringe metro, 3) medium metro, 4) small metro, 5) micropolitan, and 6) non-core rural counties.

Figures 2 and 3 depict the county and census tract level PWDs to parks in the US. Large metropolitan areas and highly urbanized neighborhoods have significantly better spatial access to parks. A comparison between the shortest distances to the nearest park and the PWDs to local nearest seven parks show that these two measures are strongly correlated, especially in large metropolitan areas. The Pearson's correlation coefficients are 0.93 for US, 0.92 for large central metro, 0.94 for large fringe metro, 0.91 for medium metro, 0.86 for small metro and micropolitan areas and 0.93 for noncore rural areas. On the other hand, there are significant differences among these two distance measures, from 0.4 miles in central metropolitan counties to 7.6 miles in rural counties (see table 2). These differences could make a significant impact on the actual population park access and use. Our PWD measure avoids the potential bias associated with the shortest distance measure to the nearest park and provides a more realistic picture of population potential spatial access to parks.

**Table 2:** Correlations between the population-weighted distances to parks

Geography	Nearest Park(miles)	Nearest seven parks(miles)	Difference(miles)	Pearson Correlation Coefficients
US	4.3	6.7	2.5	0.93
Large central metro	0.7	1.2	0.4	0.92
large fringe metro	1.9	3.0	1.0	0.94
Medium metro	4.3	6.8	2.5	0.91
Small metro	9.0	14.5	5.4	0.86
Micropolitan	9.3	15.0	5.8	0.86
Noncore	14.6	22.2	7.6	0.93

## Discussion

This article reviewed some significant bias and disadvantages associated with traditional spatial access measures of neighborhood populations to local parks and proposed a new approach. This new approach that measures potential spatial access to parks has a very flexible framework to incorporate significant differences in park location and their spatial configuration along the urban-rural continuum. It easily aggregates the potential spatial access to park measures from block level to larger geographic units (census tract, county, state and nation) with an explicit consideration of heterogeneous population distribution across the landscape. Thus, the results from this measure allow park access comparisons among different geographic settings as well as at multiple scales (cross-scale) as we demonstrated. When a local park attractiveness measure is available, it easily could be added to the current modeling framework to generate a more accurate picture of local park access, if necessary.

### The measure of potential spatial access to parks and public health intervention

A good measure of potential spatial access to parks should be able to serve as a base to inform public health intervention. Our PWDs to the seven nearest parks could not provide a complete landscape of park access and use determined by individual population characteristics (individual preferences), attractiveness of potential destinations (including park safety and quality of park facilities) and spatial structure of park distributions as suggested activity-based studies[42]. The full spectrum of these factors of park access and use is beyond the scope of this paper. But residential spatial proximity to parks is a critical determinant of park use and leisure exercise, especially in urban areas. A park use study in Los Angeles, California, shows that 81% of park users lived within one mile of the neighborhood parks[43]. A recent study shows that the use of public space, such as parks, is sensitive to distance and that the park size is more likely than other attributes to attract its users[28]. From a public health intervention perspective, park location and size are the most non-modifiable components that determine the possibility and the frequency of park use. Park attractiveness is much more modifiable compared to park location and size, especially in urbanized areas. Park size and location are usually fixed. New park creation often involves substantial social and financial efforts such that improving park attractiveness is a more realistic choice in most situations. Our potential access measure with an explicit consideration of park location and size provide a basic platform to identify the exact needs to improve park use by local residential populations and could guide further interventions. For example, the black population in high poverty neighborhoods in large central metropolitan areas has the smallest PWDs to their nearest local parks; however, park safety issues could become the major significant barrier for their use by this segment of population [21,44]. The spatial match and mismatch of sociodemographics and potential spatial accessibility to parks could directly inform local community policy makers for appropriate interventions to improve park access and use. Thus, the PWD to parks could be a useful tool for neighborhood population park access evaluation and planning interventions.

### Potential spatial access to parks, spatial cognition and destination choice set formation

The traditional measures for spatial access to parks have no consideration of spatial cognition and spatial destination choice set issues. Theoretically, residential populations could access all parks in the study areas and even in the entire US. The basic assumption is that individuals are assumed to be able to evaluate all possible alternatives (all parks in the study area) prior to making a final selection. However, spatial cognition (the way individuals process spatial information on their environment) research shows that destination choice in a spatial context should be selected from a much more compact choice set because individuals may have limited spatial knowledge of all destinations[45]. The formation of this compact choice set from a universal set of all destination alternatives remains an unsolved methodological problem [31,36]. However, most destination choice alternatives will be eliminated and will not be considered at the very first stage of individuals' decision making process, because individuals have no spatial knowledge and also have limited information processing capacity. Park choice set formulation in spatial contexts depends psychologically on the spatial cognition of individuals. The universal choice set that consists of all parks available for an individual should reduce to a small number of parks. Previous research on psychological limits of cognition of individuals confirms that seven is the number that an individual could do pair-wise comparison among all alternatives with reliable validity[33,34]. We explicitly incorporated this psychological upper limit of individual information processing to develop a more behaviorally realistic potential park access measure. We assumed that the spatial choice set formation of individuals was similar to the process that explains the aggregated spatial choice set formation of populations (a large number of individuals). This is a potential limitation. Urban neighborhood parks were often unused during substantial portions of the weekdays, and even weekends [43]. So we reasonably assumed that public parks service capacity for local resident populations would not affect their spatial accessibility. This is different from spatial access to health care services, and the ratio of service supply and demand is a critical component to evaluate their spatial accessibility [46,47].

### Spatial equity of park access and potential park access along the urban-rural continuum

Neighborhood park accessibility has been examined in a broader context of environmental justice[48]. Better park access and use promotes leisure time physical activities, such as walking and biking, and neighborhood parks also provide environmental buffers improving resident psychological and mental health as well as community social health via exposure to natural restorative environments and increasing social activities [49,50]. Parks have become critical open spaces that promote physical activity and facilitate social interaction among community residents, especially in urban areas. Thus, parks as important community resources have gained more attention from an environmental justice perspective [15,20,48,51-54]. The central concept of environmental justice related to park access is about the spatial equity of park access. However the conceptualization and measurement of park access is the critical basis for evaluating spatial accessibility of parks and its relationships to spatial distribution of populations by sociodemographics. The potential spatial access measured by PWD to parks in this paper is based on population locations and sizes as well as park locations and sizes, and provides an informative measurement tool to understand population-based spatial accessibility to parks, thus the spatial equity of park access. Our results show that spatial accessibility to parks may not be the key factor contributing to physical inactivity of minorities and population in poor urban neighborhoods in US large metropolitan areas. The safety of parks and neighborhoods may be the major barriers for population park access and use [21]. Thus, local community public health intervention strategies should focus more on how to create a safe environment for park access and use.

Potential spatial access to parks significantly increases along the urban-rural geographic continuum. Residential populations at different geographic urban-rural settings use different travel modes to access parks and have very different perceptions about the physical travel distances to parks. Most populations in suburban and rural areas use cars to access parks. Thus a simple comparison of our PWD measures along the urban-rural continuum would not be informative. Figure 3 shows census tract level relative park accessibility measured by PWD. For each county-level NCHS urban-rural category from central metropolitan to non-core rural area counties, we categorized the census tract level PWD into five groups defined by quintiles. These census tract level relative park accessibility measures are more comparable across the urban-rural continuum in the US.

## Conclusions

In conclusion, the focus of this paper was to develop a more flexible, accurate and informative modeling framework for quantifying the spatial distributions of parks in relation to population locations that can be used by local policy-makers in developing intervention strategies to increase park access and use and thus improve population physical activity. Toward this objective, our integrative modeling framework of spatial access to parks explicitly incorporates advantages of classic park access measures: the intuitiveness of direct travel cost-based measures, the flexibility of spatial interaction model-based measures, and the limited spatial access of container-based measures. In particular, the proposed conceptual framework took into account the limited psychological information processing theory in choice modeling and spatial cognition, which make it more behaviorally realistic and practical. The modeling framework was further extended to generate a population-weighted measure of spatial access to parks by applying the probability theory of trade-area analysis in marketing research. This new measure of spatial access to parks is flexible, by incorporating available local community data to improve park access measurement and by more accurately comparing park spatial access among different local residential populations. The new measure is more accurate in that it explicitly incorporates the locations and sizes of both population and parks and takes account of heterogeneous population distribution. The new measure is informative in its incorporation of different components of spatial decision processes: spatial cognition, spatial interaction and probability access. The application of this new measure for spatial equity of park access indicates that park safety, rather than park spatial accessibility, may be a major barrier to park access and use by the populations in poor urban neighborhoods [21].

Future research will focus on the linkage of the measure of spatial access to parks with physical activity outcomes from national health surveillance datasets such as the National Health and Nutrition Examination Survey (NHANES), the National Health Interview Survey (NHIS), and the Behavioral Risk Factor Surveillance System (BRFSS), to better explain the inconsistencies of the park's role in promoting physical activity among many previous local studies. We wanted to translate the potential spatial access to parks into an informative evaluation tool for health professionals and policy makers. Social-spatial equity analysis of park access within a specific urban or rural geographic setting could provide more meaningful conclusions about population spatial accessibility of parks. Furthermore, this method could be applied to quantify geographic accessibility of other types of services or destinations, such as food, alcohol, and tobacco outlets.

## Competing interests

The authors declare that they have no competing interests.

## Authors' contributions

XZ, JBH conceived the study and wrote the final version of the paper. HL prepared the mapping and participated in the draft revision. All authors have read and approved the final manuscript.

## Authors' information

The findings and conclusions in this report are those of the authors and do not necessarily represent the official position of the Centers for Disease Control and Prevention (CDC). No external funding was received for this study.

## References

[B1] Flegal KM Carroll MD Kuczmarski RJ Johnson CL Overweight and obesity in the United States: prevalence and trends, 1960-1994 International Journal of Obesity 1998 22 1 39 47 10.1038/sj.ijo.0800541 9481598

[B2] Flegal KM Carroll MD Ogden CL Curtin LR Prevalence and Trends in Obesity Among US Adults, 1999-2008 Jama-Journal of the American Medical Association 2010 303 3 235 241 10.1001/jama.2009.2014 20071471

[B3] Kuczmarski RJ Flegal KM Campbell SM Johnson CL Increasing prevalence of overweight among US adults-The National-health and nutrition examination surveys, 1960 to 1991 Jama-Journal of the American Medical Association 1994 272 3 205 211 10.1001/jama.272.3.205 8022039

[B4] Finkelstein EA Trogdon JG Cohen JW Dietz W Annual medical spending attributable to obesity: payer-and service-specific estimates Health Aff (Millwood) 2009 28 5 w822 831 10.1377/hlthaff.28.5.w822 19635784

[B5] Congressional Budget Office How does obesity in adults affect spending on health care? Washington, DC 2010

[B6] Jackson RJ The impact of the built environment on health: an emerging field Am J Public Health 2003 93 9 1382 1384 10.2105/AJPH.93.9.1382 12948946 PMC1447976

[B7] Wakefield J Fighting obesity through the built environment Environmental health perspectives 2004 112 11 A616 618 10.1289/ehp.112-a616 15289181 PMC1247493

[B8] Bedimo-Rung AL Mowen AJ Cohen DA The significance of parks to physical activity and public health: a conceptual model Am J Prev Med 2005 28 2 Suppl 2 159 168 15694524 10.1016/j.amepre.2004.10.024

[B9] Coombes E Jones AP Hillsdon M The relationship of physical activity and overweight to objectively measured green space accessibility and use Social science & medicine (1982) 2010 70 6 816 822 10.1016/j.socscimed.2009.11.020 20060635 PMC3759315

[B10] Sugiyama T Francis J Middleton NJ Owen N Giles-Corti B Associations between recreational walking and attractiveness, size, and proximity of neighborhood open spaces Am J Public Health 100 9 1752 1757 10.2105/AJPH.2009.182006 PMC2920990 20634455

[B11] Task Force on Community Preventive Services Recommendations to increase physical activity in communities Am J Prev Med 2002 22 4 Suppl 67 72 11985935 10.1016/s0749-3797(02)00433-6

[B12] Gies E The health Benefits of Parks 2007 San Francisco, CA: The Trust for Pulic Land

[B13] Kaczynski AT Henderson KA Environmental correlates of physical activity: A review of evidence about parks and recreation Leisure Sciences 2007 29 4 315 354 10.1080/01490400701394865

[B14] Kaczynski AT Potwarka LR Smale BJA Havitz ME Association of Parkland Proximity with Neighborhood and Park-based Physical Activity: Variations by Gender and Age Leisure Sciences 2009 31 2 174 191 10.1080/01490400802686045

[B15] Cutts BB Darby KJ Boone CG Brewis A City structure, obesity, and environmental justice: An integrated analysis of physical and social barriers to walkable streets and park access Social Science & Medicine 2009 69 9 1314 1322 10.1016/j.socscimed.2009.08.020 19751959

[B16] Cohen DA Ashwood JS Scott MM Overton A Evenson KR Staten LK Porter D McKenzie TL Catellier D Public parks and physical activity among adolescent girls Pediatrics 2006 118 5 E1381 E1389 10.1542/peds.2006-1226 17079539 PMC2239262

[B17] Neutens T Schwanen T Witlox F De Maeyer P Equity of urban service delivery: a comparison of different accessibility measures Environment and Planning A 2010 42 7 1613 1635 10.1068/a4230

[B18] Kwan MP Weber J Scale and accessibility: Implications for the analysis of land use-travel interaction Applied Geography 2008 28 2 110 123 10.1016/j.apgeog.2007.07.002

[B19] Kaczynski AT Potwarka LR Saelens BE Association of park size, distance, and features with physical activity in neighborhood parks American Journal of Public Health 2008 98 8 1451 1456 10.2105/AJPH.2007.129064 18556600 PMC2446450

[B20] Talen E The social equity of urban service distribution an exploration of park access in Pueblo, Colorado, and Macon, Georgia Urban Geography 1997 18 6 521 541 10.2747/0272-3638.18.6.521

[B21] Carlson SA Brooks JD Brown DR Buchner DM Racial/Ethnic differences in perceived access, environmental barriers to use, and use of community parks Preventing chronic disease 2010 7 3 A49 20394688 PMC2879981

[B22] Omer I Evaluating accessibility using house-level data: A spatial equity perspective Computers Environment and Urban Systems 2006 30 3 254 274 10.1016/j.compenvurbsys.2005.06.004

[B23] Maroko AR Maantay JA Sohler NL Grady KL Arno PS The complexities of measuring access to parks and physical activity sites in New York City: a quantitative and qualitative approach International Journal of Health Geographics 2009 8 10.1186/1476-072X-8-34 PMC2708147 19545430

[B24] Shi X Selection of bandwidth type and adjustment side in kernel density estimation over inhomogeneous backgrounds Int J Geogr Inf Sci 2010 24 5 643 660 10.1080/13658810902950625

[B25] Giles-Corti B Donovan R Holman C Factors influencing the use of physical activity facilities: results from qualitative research Health Promotion J 1996 6 1 16 21

[B26] Giles-Corti B Broomhall MH Knuiman M Collins C Douglas K Ng K Lange A Donovan RJ Increasing walking: how important is distance to, attractiveness, and size of public open space? Am J Prev Med 2005 28 2 Suppl 2 169 176 15694525 10.1016/j.amepre.2004.10.018

[B27] Giles-Corti B Donovan RJ The relative influence of individual, social and physical environment determinants of physical activity Social science & medicine (1982) 2002 54 12 1793 1812 10.1016/S0277-9536(01)00150-2 12113436

[B28] Giles-Corti B Donovan RJ Relative influences of individual, social environmental, and physical environmental correlates of walking Am J Public Health 2003 93 9 1583 1589 10.2105/AJPH.93.9.1583 12948984 PMC1448014

[B29] Hillsdon M Panter J Foster C Jones A The relationship between access and quality of urban green space with population physical activity Public health 2006 120 12 1127 1132 10.1016/j.puhe.2006.10.007 17067646

[B30] Haynes R Lovett A Sunnenberg G Potential accessibility, travel time, and consumer choice: geographical variations in general medical practice registrations in Eastern England Environment and Planning A 2003 35 10 1733 1750 10.1068/a35165

[B31] Thill JC Choice Set Formation For Destination Choice Modeling Progress in Human Geography 1992 16 3 361 382 10.1177/030913259201600303

[B32] Fotheringham A Consumer Choice and Choice Set Definition Marketing Science 1988 7 299 310 10.1287/mksc.7.3.299

[B33] Miller G The Magical Number Seven, Plus or Minus: Some limits on Our Capacity for Processing Information Psychological Review 1956 63 2 81 97 13310704

[B34] Saaty TL Ozdemir MS Why the magic number seven plus or minus two Mathematical and Computer Modelling 2003 38 3-4 233 244 10.1016/S0895-7177(03)90083-5

[B35] Meyer J A descriptive model of constrained residential search Geographic Analysis 1980 12 21 32

[B36] Pellegrini PA Fotheringham AS Lin G An empirical evaluation of parameter sensitivity to choice set definition in shopping destination choice models Papers in Regional Science 1997 76 2 257 284

[B37] Kareev Y Seven (indeed, plus or minus two) and the detection of correlations Psychological Review 2000 107 2 397 402 10789204 10.1037/0033-295x.107.2.397

[B38] Huff D Defining and Estimating a Trading Area The Journal of Marketing 1964 3 34 38

[B39] Apparicio P Abdelmajid M Riva M Shearmur R Comparing alternative approaches to measuring the geographical accessibility of urban health services: Distance types and aggregation-error issues International Journal of Health Geographics 2008 7 10.1186/1476-072X-7-7 PMC2265683 18282284

[B40] Haynes R Jones AP Sauerzapf V Zhao H Validation of travel times to hospital estimated by GIS Int J Health Geogr 2006 5 40 10.1186/1476-072X-5-40 16984650 PMC1586189

[B41] Cascetta E Papola A Dominance among alternatives in random utility models Transportation Research Part a-Policy and Practice 2009 43 2 170 179 10.1016/j.tra.2008.10.003

[B42] Sivakumar A Bhat CR Comprehensive, unified framework for analyzing spatial location choice Transportation Research Record 2007 2003 103 111 10.3141/2003-13

[B43] Cohen DA Sehgal A Williamson S Sturm R McKenzie TL Lara R Lurie N Park use and physical activity in a sample of public parks in the city of Los Angeles 2006 Santa Monica, CA: RAND Corporation

[B44] Leslie E Cerin E Kremer P Perceived Neighborhood Environment and Park Use as Mediators of the Effect of Area Socio-Economic Status on Walking Behaviors J Phys Act Health 2010 7 6 802 810 21088312 10.1123/jpah.7.6.802

[B45] Fotheringham AS Curtis A Regularities in spatial information processing: Implications for modeling destination choice Professional Geographer 1999 51 2 227 239 10.1111/0033-0124.00159

[B46] Khan AA An integrated approach to measuring potential spatialaccess to health-care services Socio-Economic Planning Sciences 1992 26 4 275 287 10.1016/0038-0121(92)90004-O 10123094

[B47] Wang FH Luo W Assessing spatial and nonspatial factors for healthcare access: towards an integrated approach to defining health professional shortage areas Health & Place 2005 11 2 131 146 10.1016/j.healthplace.2004.02.003 15629681

[B48] Boone CG Buckley GL Grove JM Sister C Parks and People: An Environmental Justice Inquiry in Baltimore, Maryland Annals of the Association of American Geographers 2009 99 4 767 787 10.1080/00045600903102949

[B49] Gies E The Health Benefits of Parks 2006 San Francisco, CA: The Trust for Public Land

[B50] Cutts BB Darby KJ Boone CG Brewis A City structure, obesity, and environmental justice: an integrated analysis of physical and social barriers to walkable streets and park access Social science & medicine (1982) 2009 69 9 1314 1322 10.1016/j.socscimed.2009.08.020 19751959

[B51] Byrne J Wolch J Zhang J Planning for environmental justice in an urban national park Journal of Environmental Planning and Management 2009 52 3 365 392 10.1080/09640560802703256

[B52] Wolch J Wilson JP Fehrenbach J Parks and park funding in Los Angeles: An equity-mapping analysis Urban Geography 2005 26 1 4 35 10.2747/0272-3638.26.1.4

[B53] Byrne J Wolch J Nature, race, and parks: past research and future directions for geographic research Progress in Human Geography 2009 33 6 743 765 10.1177/0309132509103156

[B54] Taylor WC Floyd MF Whitt-Glover MC Brooks J Environmental justice: a framework for collaboration between the public health and parks and recreation fields to study disparities in physical activity J Phys Act Health 2007 4 Suppl 1 S50 63 17672223 10.1123/jpah.4.s1.s50

